# Trauma from occlusion — An orthodontist’s perspective

**DOI:** 10.4103/0972-124X.70838

**Published:** 2010

**Authors:** R. Saravanan, Prajeeth J. Babu, P. Rajakumar

**Affiliations:** *Department of Orthodontics, Thai Moogambigai Dental College and Hospital, Chennai, Tamil Nadu, India*

## Abstract

Orthodontic therapy has a big role in the treatment and prevention of malpositions. The signs and symptoms experienced by patients with occlusal trauma are mobility of teeth, temperomandibular joint pain, pain on mastication and periodontal disease. Early diagnosis, proper treatment plan and correction of malocclusion can lead to a successful outcome. Lack of awareness of orthodontic treatment in patients with occlusal trauma can even lead to loss of tooth structure.

## INTRODUCTION

Occlusal trauma has been defined as injury to the periodontium resulting from occlusal forces that exceeds the reparative capacity of the attachment apparatus. Trauma from occlusion refers to tissue injury due to distorted occlusion. An occlusion that produces such injury is called a traumatic occlusion. Acute trauma from occlusion can result from an abrupt occlusal impact on bitten objects, restorations or prosthetic appliances that interfere with or alter the occlusal forces on teeth.

Chronic trauma from occlusion is more common than acute trauma and develops from gradual changes in occlusion produced by tooth wear, drifting movement, extrusion of teeth, combined with parafunctional habits such as bruxism and clenching.

Trauma from occlusion is considered the primary etiological factor, and the only local alteration to which a tooth is subjected is from occlusion. Secondary trauma from occlusion occurs when the adaptive capacity of the tissues to withstand occlusal forces is impaired by bone loss resulting from marginal inflammation. The periodontium becomes more vulnerable to injury, and previously well-tolerated occlusal forces become traumatic.

The diagnosis and assessment (flowchart) of occlusal trauma is not merely made based on a single examination, due to the necessarily progressive nature of injury. Orthodontic correction is usually restricted to cases where tooth malpositions are the prime cause of trauma. There are certain additional factors such as morphology, prognosis of the teeth involved, direction and magnitude of movement required that will influence the decision of whether or not orthodontic tooth movement is indicated.

**Figure:**
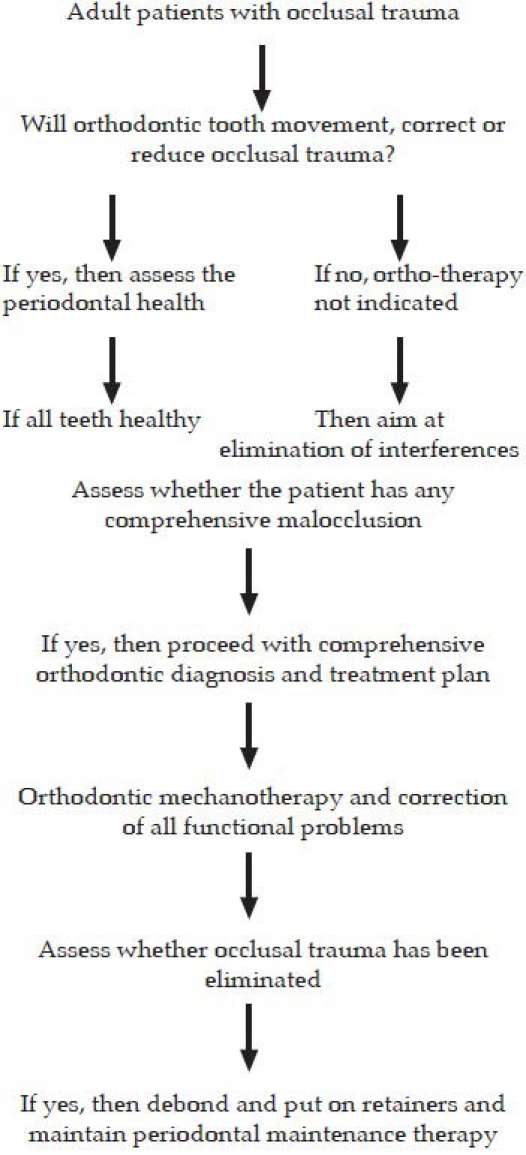
Flow chart for assessment of occlusal trauma

Limited orthodontic mechanotherapy assumes that either the traumatic lesion is the only evidence of a malocclusion or that the patients have been informed of the mode of management aimed at comprehensive treatment of malocclusion and have rejected the same.

Before orthodontic appliances are removed, the clinician should ensure that occlusal interferences are eliminated. This assessment should be made in both static and functional excursion. Investigations that may aid this decision may include visual inspection using articulating paper, occlusion, mobility assessment, radiographs and the use of the lately developed computer-aided occlusal evaluation systems.

Once traumatic occlusion has been eliminated via tooth movement, and other treatment goals are obtained the patients appliance can be debonded and retention phase started. Retention should be custom-designed for each patient taking into account the nature of the initial malocclusion and periodontal status of the patient.

## CONCLUSION

This article aims to emphasize the role of early detection of occlusal trauma and thereafter providing orthodontic treatment to prevent further damage to periodontium and loss of dental structures. Many a times, the role of occlusion in trauma from occlusion is neglected, which can hamper early diagnosis and intervention.

